# Supplementary material for: Organic Nanobowls Modified Thin Film Composite Membrane for Enhanced Purification Performance toward Different Water Resources
Source: Membranes (Basel). 2021 May 10;11(5):350. doi: 10.3390/membranes11050350 (PMC8151631; doi:10.3390/membranes11050350)
Supplement: Supplementary file 1 [file membranes-11-00350-s001.zip › membranes-1208917-supplementary.pdf]

membranes

Supplementary Materials

Organic Nanobowls Modified Thin Film Composite Membrane for Enhanced Purification Performance toward Different Water Resources

Changjin Ou [1] ,Sisi Li [1],Zhongyi Wang [1] ,Juan Qin [1] ,Qian Wang [2] ,Zhipeng Liao [1, 3, *] ,Jiansheng Li [3, *]

[1] Nantong Key Laboratory of Intelligent and New Energy Materials, School of Chemistry and Chemical Engineering, Nantong University, Nantong, 222100, China ocj1987@ntu.edu.cn (C.O); LISISI4408@163.com (S.L.); wzy564872358@163.com (Z.W.); qinjuan880816@ntu.edu.cn (J.Q.).

[2] School of Materials Science and Chemical Engineering, Xi'an Technological University, Xi'an, 710021, China; drifting_leaf@126.com

[3] Key Laboratory of New Membrane Materials, Ministry of Industry and Information Technology, School of Environment and Biological Engineering, Nanjing University of Science and Technology, Nanjing, 210094, China.

* Correspondence: zhpengliao@163.com (Z.L.); lijsh@njust.edu.cn (J.L.)

Table S1. Comparisons of removal performances of various composite membranes toward seawater.

	Ca^{2+} (%)	Mg^{2+} (%)	Na^{+} (%)	K^{+} (%)	SO_4^{2-} (%)
TFC	22.91 ± 0.73	22.3 ± 0.58	15.71 ± 0.23	6.28 ± 0.45	76.92 ± 1.71
TFN	34.44 ± 0.32	36.4 ± 3.06	14.73 ± 2.1	8.34 ± 1.02	76.99 ± 2.56
CM	27.8 ± 0.12	27.25 ± 0.43	22.2 ± 0.17	10.39 ± 0.25	78.17 ± 0.17
	NO_3^- (%)	Cl^- (%)	TOC (%)	pH	Viscosity (cP)
TFC	14.44 ± 0.8	5.2 ± 0.52	82.61 ± 0.74	7.95 ± 0.21	Nondetected
TFN	14.58 ± 1.92	10.72 ± 0.62	81.29 ± 3.95	7.76 ± 0.14	Nondetected
CM	14.57 ± 0.31	7.46 ± 0.04	85.16 ± 0.38	7.82 ± 0.08	Nondetected

The initial seawater conditions: Ca^{2+} of 0.22 g/L, Mg^{2+} of 0.61 g/L, Na^{+} of 4.01 g/L, K^{+} of 0.48 mg/L, SO_4^{2-} of 3.71 g/L, NO_3^- of 15.29 mg/L, Cl^- of 10.58 g/L, TOC of 21.49 mg/L, pH of 8.02 and Viscosity of 1.50 cP

Table S2. Comparisons of removal performances of various composite membranes toward tap water.

	Ca^{2+} (%)	Mg^{2+} (%)	SO_4^{2-} (%)	Cl^- (%)	TOC (%)	pH
TFC	79.71 ± 0.85	68.53 ± 0.42	74.52 ± 0.78	49.54 ± 1.07	84.09 ± 2.9	6.78 ± 0.29
TFN	84.36 ± 2.11	76.38 ± 2.17	77.54 ± 1.24	57.49 ± 1.70	83.44 ± 0.25	6.94 ± 0.07
CM	83.49 ± 0.37	72.51 ± 0.67	77.5 ± 0.36	55.16 ± 0.32	83.98 ± 0.27	6.95 ± 0.09

The initial tap water conditions: Ca^{2+} of 43.80 mg/L, Mg^{2+} of 13.50 mg/L, SO_4^{2-} of 36.67 mg/L, Cl^- of 47.05 mg/L, TOC of 3.32 mg/L and pH of 6.64.

Table S3. Comparisons of removal performances of various composite membranes toward municipal effluent.

	Ca^{2+} (%)	Mg^{2+} (%)	SO_4^{2-} (%)	Cl^- (%)	TOC (%)	pH
TFC	68.91 ± 1.43	76.74 ± 0.89	64.3 ± 1.13	74.41 ± 0.58	83.04 ± 1.36	6.86 ± 0.07
TFN	72.68 ± 0.73	79.64 ± 2.65	64.09 ± 0.58	74.15 ± 0.37	83.23 ± 0.93	6.87 ± 0.04
CM	69.44 ± 0.25	79.08 ± 0.43	66.05 ± 0.33	75.67 ± 0.10	83.71 ± 0.29	7.00 ± 0.02

The initial municipal effluent water conditions: Ca^{2+} of 70.61 mg/L, Mg^{2+} of 16.6 mg/L, SO_4^{2-} of 33.95 mg/L, Cl^- of 39.77 mg/L, TOC of 35.45 mg/L and pH of 6.15.

Table S4. Comparisons of removal performances of various composite membranes toward Ni^{2+} wastewater.

	Ni^{2+} (%)	Ca^{2+} (%)	Mg^{2+} (%)	SO_4^{2-} (%)	Cl^- (%)	TOC (%)	pH
TFC	90.92 ± 0.53	82.15 ± 0.68	61.21 ± 1.54	94.47 ± 0.59	83.71 ± 1.38	83.31 ± 1.61	6.06 ± 0.17
TFN	95.53 ± 1.88	93.07 ± 0.24	75.67 ± 1.89	94.43 ± 0.84	86.61 ± 0.82	81.81 ± 1.34	6.13 ± 0.26
CM	94.04 ± 0.17	91.04 ± 0.14	70.23 ± 0.21	95.02 ± 0.33	85.47 ± 0.41	84.59 ± 0.43	6.29 ± 0.04

The initial Ni^{2+} water conditions: Ni^{2+} of 272.45 mg/L, Ca^{2+} of 19.51 mg/L, Mg^{2+} of 14.3 mg/L, SO_4^{2-} of 1712.52 mg/L, Cl^- of 703.94 mg/L, TOC of 327.75 mg/L and pH of 2.68; The Ni^{2+} water conditions after the adjust of pH: Ni^{2+} of 203.21 mg/L, Ca^{2+} of 11.43 mg/L, Mg^{2+} of 13.2 mg/L, SO_4^{2-} of 1947.80 mg/L, Cl^- of 599.31 mg/L, TOC of 127.22 mg/L and pH of 5.94.

Table S5. Comparisons of removal performances of various composite membranes toward Cr^{3+} wastewater.

	Cr^3 (%)	Ca^{2+} (%)	Mg^{2+} (%)	SO_4^{2-} (%)	Cl^- (%)	TOC (%)	pH
TFC	98.62 ± 0.19	73.68 ± 0.87	58.06 ± 1.79	94.60 ± 0.44	59.41 ± 1.19	88.14 ± 1.33	6.17 ± 0.13
TFN	99.35 ± 0.24	77.29 ± 1.92	64.7 ± 1.53	95.30 ± 0.11	67.69 ± 1.04	88.76 ± 1.19	6.34 ± 0.09
CM	99.13 ± 0.13	77.3 ± 0.46	62.16 ± 0.38	95.07 ± 0.33	64.95 ± 0.34	90.11 ± 0.44	6.76 ± 0.09

The initial Cr^{3+} water conditions: Cr^{3+} of 3361.72 mg/L, Ca^{2+} of 20.48 mg/L, Mg^{2+} of 7.97 mg/L, SO_4^{2-} of 21142.58 mg/L, Cl^- of 765.53 mg/L, TOC of 1369.32 mg/L and pH of 1.53; The Cr^{3+} water conditions after the adjust of pH: Cr^{3+} of 1266.95 mg/L, Ca^{2+} of 10.18 mg/L, Mg^{2+} of 2.51 mg/L, SO_4^{2-} of 18156.99 mg/L, Cl^- of 557.05 mg/L, TOC of 969.88 mg/L and pH of 5.73.

Table S6. Comparisons of removal performances of various composite membranes toward Zn^{2+} wastewater.

	Zn^{2+} (%)	Ca^{2+} (%)	Mg^{2+} (%)	SO_4^{2-} (%)	Cl^- (%)	TOC (%)	pH
TFC	98.72 ± 0.29	74.24 ± 1.72	58.30 ± 1.92	91.46 ± 0.61	75.43 ± 1.78	82.00 ± 0.68	6.46 ± 0.11
TFN	99.28 ± 0.20	79.67 ± 0.89	71.40 ± 1.23	91.59 ± 1.12	79.10 ± 0.64	83.25 ± 0.49	6.66 ± 0.08
CM	98.99 ± 0.09	75.85 ± 0.31	68.48 ± 0.34	91.73 ± 0.20	77.96 ± 0.06	84.37 ± 0.15	6.7 ± 0.05

The initial Zn^{2+} water conditions: Zn^{2+} of 2908.37 mg/L, Ca^{2+} of 97.30 mg/L, Mg^{2+} of 25.30 mg/L, SO_4^{2-} of 1195.82 mg/L, Cl^- of 5023.85 mg/L, TOC of 69.6 mg/L and pH of 2.56; The Zn^{2+} water conditions after the adjust of pH: Zn^{2+} of 2070.90 mg/L, Ca^{2+} of 48.97 mg/L, Mg^{2+} of 12.66 mg/L, SO_4^{2-} of 505.21 mg/L, Cl^- of 4755.93 mg/L, TOC of 37.1 mg/L and pH of 6.3.

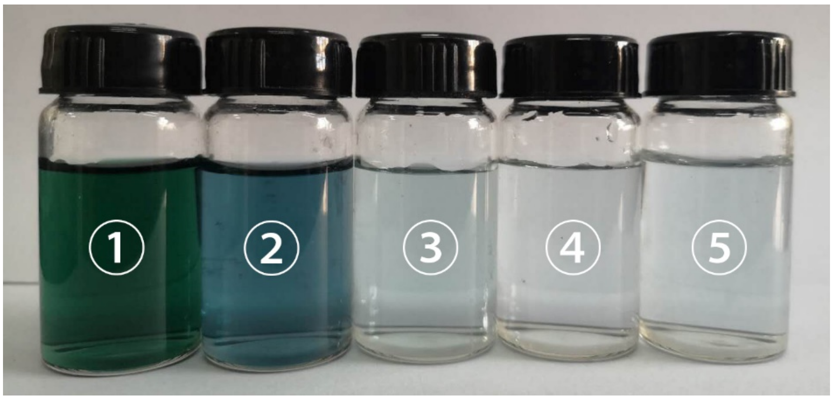

Figure S1. Comparisons of the photograph of Cr^{3+} wastewater before and after treatment: ① origin wastewater, ② after pH adjustment; effluent of ③ TFC, ④TFN and ⑤ CM membranes.

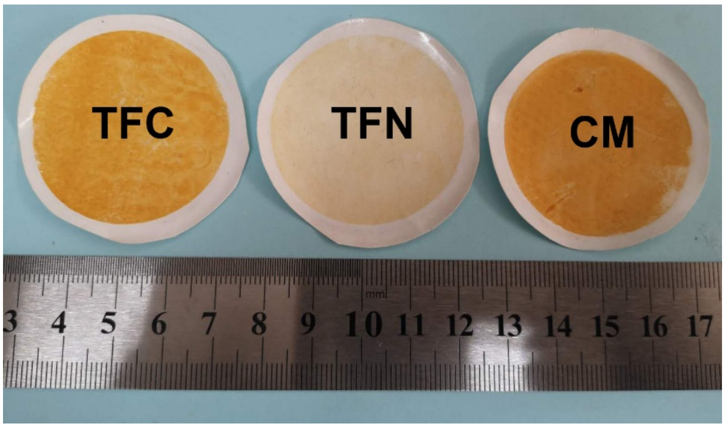

Figure S2. Photograph of fouling extent of composite membranes of separating Zn^{2+} wastewater.
